# Finding Top-*k* Covering Irreducible Contrast Sequence Rules for Disease Diagnosis

**DOI:** 10.1155/2015/353146

**Published:** 2015-03-10

**Authors:** Yuhai Zhao, Yuan Li, Ying Yin, Gang Sheng

**Affiliations:** ^1^College of Information Science and Engineering, Northeastern University, Shenyang, Liaoning 110819, China; ^2^Software Center, Northeastern University, Shenyang, Liaoning 110004, China

## Abstract

Diagnostic genes are usually used to distinguish different disease phenotypes. Most existing methods for diagnostic genes finding are based on either the individual or combinatorial discriminative power of gene(s). However, they both ignore the common expression trends among genes. In this paper, we devise a novel sequence rule, namely, top-*k* irreducible covering contrast sequence rules (Top*k*IRs for short), which helps to build a sample classifier of high accuracy. Furthermore, we propose an algorithm called MineTop*k*IRs to efficiently discover Top*k*IRs. Extensive experiments conducted on synthetic and real datasets show that MineTop*k*IRs is significantly faster than the previous methods and is of a higher classification accuracy. Additionally, many diagnostic genes discovered provide a new insight into disease diagnosis.

## 1. Introduction

It has been proved that many diseases are closely related with genes [1–3]. In bioinformatics, such genes are called* diagnostic genes*. Capturing these genes is an important task, which helps in diagnosis, prediction, and treatment of diseases [4].

According to biological theory, only a small number of genes are directly related with a certain disease [5]. Biologists always want to exploit fewer genes to provide higher disease prediction accuracy. In practice, how to pick out these diagnostic genes to distinguish different disease phenotypes from a massive amount of gene expression data is often an intractable problem.

Many studies have shown that contrast rules are very promising for this problem. Contrast rules refer to the rules that frequently appear in one class but rarely in other classes, denoted as *X* → *C*, where *X* represents the diagnostic genes and *C* represents a certain disease. Most of such methods can be divided into two categories, that is, single discrimination based [6] and combinatorial discrimination based [7]. The former evaluates every gene according to their individual discriminative power to the target classes and then selects top-ranked genes. The latter often models the problem as a subset search problem and focuses on the combinatorial discriminative power of a set of genes. However, neither of the two exploits the relationship among genes such that some important diagnostic genes may be missed.

In this paper, we tackle the problem by utilizing the order relationship among genes. Below is a real example for an immediate comprehension to our basic idea.

**Example 1 .**

**Figure 1:**
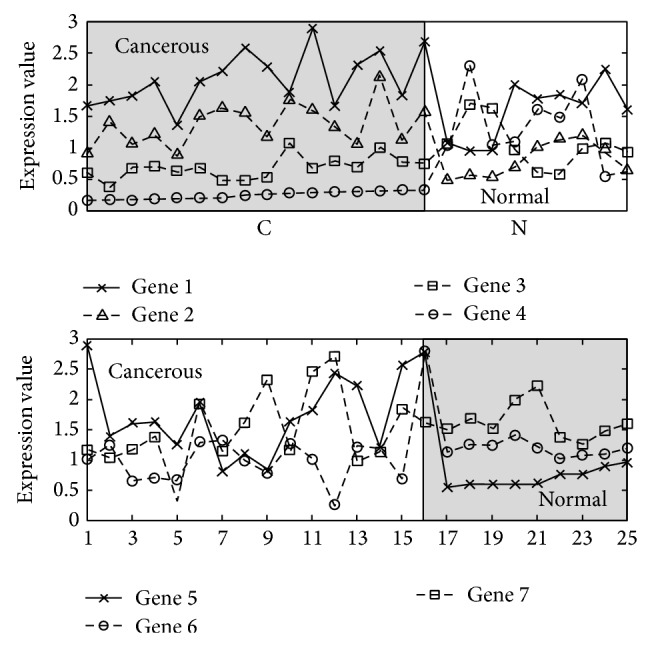
A real example from the prostate cancer dataset.

Figure 1 consists of two subfigures. In the top subfigure, 4 genes are expressed over 25 samples. Samples 1~16 are cancerous (labeled as “*C*”) and samples 17~25 are normal (labeled as “*N*”). In the bottom subfigure, another set of 3 genes is expressed over the same set of samples. The existing singleton or combination discriminability-based methods cannot distinguish the two phenotypes. Since most genes are of similar average expression values in the two phenotypes, they will not be selected by the singleton approach. Moreover, all genes are expressed in both phenotypes. Thus, the combination approach based on the cooccurrence of genes will not select them either. Both of the methods ignore the hidden interrelation among genes. In the top subfigure, the gene order over the samples of cancerous phenotype “*C*” is always *gene*_4_≺*gene*_3_≺*gene*_2_≺*gene*_1_. Such order is disturbed in normal phenotype “*N*”. In the bottom subfigure, the gene order in normal phenotype “*N*” is *gene*_5_≺*gene*_6_≺*gene*_7_, while in cancerous phenotype “*C*” such order does not exist. Based on the ordered expression values, the disease phenotypes (the two shadowed “blocks”) are well identified.

Example 1 indicates that* contrast sequence rules* may be a promising solution to the mentioned problem. Another advantage of incorporating the sequence rule into diagnostic gene finding is that we may obtain higher disease prediction accuracy by fewer genes. This is intuitively because the order contains both individual information and combinatorial information. In [8], we proposed a contrast sequence rules mining algorithm, namely, NRMINER, and showed its effectiveness and efficiency. However, there are still some issues demanding a further consideration.

Given *n* genes, there is up to 2^*n*^ subsets of genes. Moreover, each subset of *i* genes corresponds to *i*! permutations. Thus, the number of contrast sequence rules is at least ∑_*i*=1_^*n*^(*C*_*n*_^*i*^ · *i*!) ≫ *n*! in theory. On one hand, massive rules pose a crucial challenge for biologists to interpret and validate the results. On the other hand, this may take too much time such that the proposed method is not practically feasible. In practice, we often need only a small set of representative contrast sequence rules instead of all the rules. This is also the so-called* top-k problem* in database and data mining communities. Accordingly, the goal of this paper is to discover* top-k covering irreducible contrast sequence rules* (*TopkIRs* for short) from a given gene expression dataset.

Compared with the existing methods, our contributions in this paper are claimed as follows.

1. We propose the concept of top-*k* covering irreducible contrast sequence rule, which greatly reduces the burden for biologist to interpret and validate the results and practically enables an efficient diagnostic gene finding method.
2. We devise the criteria of ranking irreducible contrast sequence rules. Based on the criteria, we can pick out shorter and fewer but more representative rules to build classifier with higher classification accuracy.
3. We develop a novel algorithm called MineTop*k*IRs to directly discover top-*k* covering irreducible contrast sequence rules without postprocess. As we know, few works address this problem in the context of sequence mining.

The rest of this paper is organized as follows. In Section 2, we introduce some preliminaries and give our problem definition.  Section 3 introduces the criteria of ranking rules.  Section 4 details the MineTop*k*IRs algorithm.  Section 5 includes the experimental results and analysis. Finally, Section 6 concludes this paper.

## 2. Preliminary

In this section, we first introduce some basic concepts useful for further discussion and then formalize the problem to be addressed in this paper.

### 2.1. Basic Concepts

A microarray dataset *D* is an *m* × *n* matrix, with *m* samples *S* = {*s*_1_, *s*_2_,…, *s*_*m*_} and *n* genes *G* = {*g*_1_, *g*_2_,…, *g*_*n*_}. A real value *d*_*ij*_ in *D* represents the expression value of gene *g*_*j*_ on sample *s*_*i*_. An example microarray dataset of 7 genes and 6 samples is shown in Table 1, where the last column lists the class label for each sample.

As mentioned, we want to tackle the problem from the gene order perspective. Accordingly, we propose the EWave model, a sequence model to represent the gene expression data. Next are some necessary concepts.

**Definition 2 .**

Given an expression matrix *D* of a sample set, *S* = {*s*_1_, *s*_2_,…, *s*_*m*_}, and a gene set, *G* = {*g*_1_, *g*_2_,…, *g*_*n*_}, if for a grouping threshold *δ*, *δ* ≥ 0, and some sample *s*_*i*_ ∈ *S*, there exists a subset, *G*′, of genes holding both (1) and (2), we say *G*′ is an equivalent dimension group, or an EDG in short, of the sample *s*_*i*_:

(1) $$\begin{array}{c} \underset{g_{j},g_{j^{\prime }}\in G^{\prime }}{\max}\left|d_{ij}-d_{ij^{\prime }}\right|<\delta \times \underset{g_{j}\in G^{\prime }}{\min}d_{ij}, \end{array}$$

(2) $$\begin{array}{c} \forall g_{i},g_{j}\in G^{\prime },\;\underset{g_{j^{\prime }}\in G^{\prime }}{\min}\left|{d_{i}}_{j}-d_{ij^{\prime }}\right|<\underset{g_{j^{\prime \prime }}\in \left(G-G^{\prime }\right)}{\min}\left|d_{ij}-{d_{i}}_{j^{\prime \prime }}\right|. \end{array}$$

Specifically, we call a gene satisfying (1) but excluded from an EDG by (2) a “breakpoint.” The method of creating EDGs is detailed in [8]. It is worthy to note that no order is considered in an EDG, where the expression values have no significant differences.

**Figure 2:**
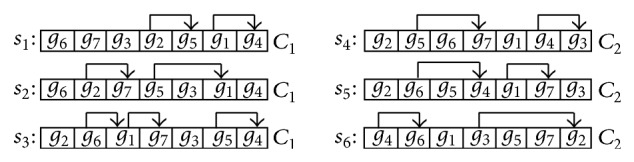
The EWave model of data in Table 1.

An EWave model can be used to represent the sequences of EDGs.  Figure 2 shows the EWave model corresponding to the running example in Table 1, where *δ* = 0.5. In each row *i* of an EWave model, all genes are increasingly ordered according to their expression values on sample *s*_*i*_, and the pointer pointing from *g*_*a*_ to *g*_*b*_ indicates an EDG starting at *g*_*a*_ and ending at *g*_*b*_. We omit the pointers pointing to a gene itself.

Different from the other traditional sequence-like data, since the overlap among different EDGs is allowed in the EWave model, a gene in an EDG can also belong to several other EDGs at the same time. Given a sample *s*_*i*_ and a gene *g*_*a*_, the sequence of EDGs of *s*_*i*_ is denoted as $_*i*_. Then, we call the index of the first EDG in $_*i*_ containing *g*_*a*_ the head position of *g*_*a*_ with respect to *s*_*i*_ and the index of the last EDG in $_*i*_ containing *g*_*a*_ the tail position of *g*_*a*_ with respect to *s*_*i*_, denoted as *H*_*i*_(*g*_*a*_) and *T*_*i*_(*g*_*a*_), respectively.

**Example 3 .**

For *s*_3_ in Figure 2, the head position of *g*_1_,  *H*_3_(*g*_1_), is 2, and the last position of *g*_1_, *T*_3_(*g*_1_), is 3.

**Definition 4 .**

Let $ be a sequence of EDGs in an EWave model, where the order of genes is *g*_*i*_1__ *g*_*i*_2__,…, *g*_*i*_*n*__. Then, we call a gene sequence *𝒢* = *h*_*j*_1__ *h*_*j*_2__,…, *h*_*j*_*t*__ is contained by $, denoted as *h*⊑$, if there exist the integers 1 ≤ *k*_1_ ≤ *k*_2_ ≤ ⋯≤*k*_*t*_ ≤ *n* such that *h*_*j*_1__ = *g*_*i*_*k*1__, *h*_*j*_2__ = *g*_*i*_*k*2__,…, *h*_*j*_*t*__ = *g*_*i*_*kt*__. Further, we refer to the gene sequence *𝒢*, where any pair of genes are not in the same EDG, as a* significant chain*.

**Example 5 .**

In Figure 2, *g*_6_ *g*_7_ *g*_5_ is a significant chain of $_1_, but *g*_6_ *g*_2_ *g*_5_ is not, since *g*_2_ and *g*_5_ coexist in the same EDG.

As mentioned above, we aim to capture the difference among different sample phenotypes from a sequence point of view. Thus, the benefit of EWave model has two aspects. On one hand, not only the gene expression data are very noisy, but also sometimes the gene expression values are very close. If we only consider the significant chain, the difference between genes is large enough so that the difficulty to determine the order among genes is overcome. On the other hand, the high dimension of gene expression data is largely reduced at the same time. Next, we introduce some concepts related with the contrast sequence rule under the EWave model.

**Definition 6 .**

Let *D* be EWave modeled gene expression data. Then, for a given sequence rule *γ*, denoted as *X* → *C*, where *X* is a significant chain and *C* is a given class label, the support of *γ* is defined as the number of the sequences of EDGs in *D* containing *XC*, denoted as supp(*γ*) and the sample support set of *γ* denoted as *R*(*γ*). The confidence of *γ* is defined as the ratio of the number of the sequences of EDGs containing *XC* to that of the sequence of EDGs containing *X*, denoted as conf(*γ*) = supp(*XC*)/supp(*X*).

**Example 7 .**

In Figure 2, let *γ* be the rule *g*_7_ *g*_4_ → *C*_1_. Then, *R*(*γ*) = {*s*_1_, *s*_2_, *s*_3_} and supp(*γ*) = 3. Further, since supp(*g*_7_ *g*_4_) = 4, conf(*γ*) = 3/4 = 75%.

**Definition 8 .**

Let *D* be an EWave modeled gene expression dataset and *C* a specified class label. RG = {*X*_*i*_ → *C*∣∃ *s* ∈ *D*, *X*_*i*_⊑$} is a* rule group* with antecedent support set *R* and consequent *C*, iff (1)  ∀*X*_*i*_ → *C* ∈ RG,  *R*(*X*_*i*_) = *R* and (2)  ∀*R*(*X*_*i*_) = *R*, *X*_*i*_ → *C* ∈ RG.

**Example 9 .**

In Figure 2, *R*(*g*_6_ *g*_7_ *g*_3_ *g*_4_) = *R*(*g*_6_ *g*_7_ *g*_4_) = *R*(*g*_6_ *g*_3_ *g*_4_) = *R*(*g*_7_ *g*_3_ *g*_4_) = *R*(*g*_3_ *g*_4_) = {*s*_1_, *s*_2_, *s*_3_}. Thus, they make up a rule group RG = {*g*_6_ *g*_7_ *g*_3_ *g*_4_ → *C*_1_, *g*_6_ *g*_7_ *g*_4_ → *C*_1_, *g*_6_ *g*_3_ *g*_4_ → *C*_1_, *g*_7_ *g*_3_ *g*_4_ → *C*_1_, *g*_3_ *g*_4_ → *C*_1_} with antecedent support set {*s*_1_, *s*_2_, *s*_3_} and a specified class label *C*_1_.

In this paper, we want to use the contrast sequence rules to distinguish the sample phenotypes. However, the number of contrast sequence rules in the dataset is prohibitively large, and most of them are redundant. Discovering all contrast sequence rules is inefficient and trivial. Thus, we propose the concept of irreducible contrast sequence rule, which is more concise and representative.

**Definition 10 .**

Let *D* be an EWave modeled gene expression dataset. A sequence rule *γ* in the form of *X* → *C* is called a contrast sequence rule if supp(*γ*) and conf(*γ*) are no less than the minimum support threshold *α* and the confidence threshold *β*, respectively, where *X* is a sequence and *C* is a class label.

**Example 11 .**

Suppose *α* = 3 and *β* = 90%. Then, *γ*: *g*_6_ *g*_7_ *g*_3_ *g*_4_ → *C*_1_ in Figure 2 is a contrast sequence rule since supp(*γ*) = 3 ≥ *α* and conf(*γ*) = 100% > *β*.

**Definition 12 .**

For any given contrast sequence rule *γ*: *X* → *C* of conf(*γ*) = *β*, we call it an irreducible contrast sequence rule if any of *X*′ → *C*  (*X*′⊑*X*) has conf(*X*′ → *C*) < *β*. In other words, any subrule of a contrast sequence rule *γ* should not be a contrast sequence rule.

**Example 13 .**

*γ*: *g*_6_ *g*_7_ *g*_3_ *g*_4_ → *C*_1_ in Figure 2 is not an irreducible contrast sequence rule since there exists a subrule of *γ*, say *γ*′: *g*_6_ *g*_7_ *g*_4_ → *C*_1_, such that conf(*γ*′) ≥ conf(*γ*) = 100%.

**Definition 14 .**

Given *D*, an EWave modeled gene expression dataset, the top-*k* covering irreducible contrast sequence rules for a sample *s*_*i*_ is the set of rules {*γ*_*s*_*i*_,*j*_}  (1 ≤ *j* ≤ *k*), where the antecedent of *γ*_*s*_*i*_,*j*_ is contained by *s*_*i*_, ∀*x*, *y* ∈ *k*, *R*(*γ*_*s*_*i*_,*x*_) ≠ *R*(*γ*_*s*_*i*_,*y*_) and there exists no rule *γ*′,  *γ*′ ∉ {*γ*_*s*_*i*_,*j*_} such that *γ*′ can substitute any rule in {*γ*_*s*_*i*_,*j*_} based on the rule priority. For brevity, we will use the abbreviation Top*k*IRs to refer to top-*k* covering irreducible contrast sequence rules for each sample.

**Example 15 .**

Suppose *k* = 2. Then, for sample *s*_1_ in Figure 2, the top-*k* covering irreducible contrast sequence rules is the set of rules {*γ*_*s*_1_,1_: *g*_7_ *g*_5_ → *C*_1_, *γ*_*s*_1_,2_: *g*_3_ *g*_4_ → *C*_1_}. This is because (1)  *s*_1_ ∈ *R*(*γ*_*s*_1_,1_) and *s*_1_ ∈ *R*(*γ*_*s*_1_,2_), (2) both *γ*_*s*_1_,1_ and *γ*_*s*_1_,2_ are irreducible contrast sequence rules, and (3) there is no other rule *γ*′ which can substitute *γ*_*s*_1_,1_ or *γ*_*s*_1_,2_ due to conf(*γ*_*s*_1_,1_) = conf(*γ*_*s*_1_,2_) = 100%.

### 2.2. Problem Description

Given (1) a gene expression dataset *D* where each sample is attached with a unique class label, (2) the equivalent threshold *δ*, (3) the minimum support threshold *α*, and (4) the confidence threshold *β*, the problem is to efficiently discover the set of top-*k* covering irreducible contrast sequence rules for each sample.

## 3. Criteria of Ranking Rules

In this section, we introduce the criteria of ranking rules. In order to evaluate the (dis)similarity between sequences, we propose the concept of projection distance which is more suitable for EWave modeled gene expression data. The reason is that projection distance takes into account not only the difference on the same position between two sequences but also the displacement between the two items.

Assume *o*_*i*_ is a gene sequence and $ is the gene sequence corresponding to sample *s*, the* projection* of *o*_*i*_ on $, denoted as *o*_*i*_∣*s*, refers to the sequence of all elements in *o*_*i*_, permuted according to their relative orders in $. Further, if a pair of items in *o*_*i*_, denoted as (*x*, *y*), has the reversal relative order in *o*_*i*_∣*s*, we call it* a reverse pair*. Then, for an item *x*, if it is at the *k*th locus in *o*_*i*_ and at the *j*th locus in *o*_*i*_∣*s*, we call |*k* − *j*| the* displacement* of *x* between *o*_*i*_ and *o*_*i*_∣*s*, denoted as dist_*x*_(*o*_*i*_, *s*).

**Definition 16 .**

Given a gene sequence *o*_*i*_ and the sequence $ corresponding to sample *s*, the* projection distance* between *o*_*i*_ and *o*_*i*_∣*s* is defined by the following formula:

(3) $$\begin{array}{c} \text{PD}\left(o_{i},o_{i}\;|\;s\right)=\underset{\begin{array}{c} x,y\in o_{i}\\ x\neq y \end{array}}{\sum }\phi \left(x,y\right)\left[\text{dis}{\text{t}}_{x}\left(o_{i},s\right)+\text{dis}{\text{t}}_{y}\left(o_{i},s\right)\right], \end{array}$$

where *ϕ*(*x*, *y*) is a Boolean function expressed as *ϕ*(*x*, *y*) = 1, if (*x*, *y*) is a reversal pair; otherwise, *ϕ*(*x*, *y*) = 0.

Now, we adopt a similarity function defined based on the concept of projection distance (or simply PD) to identify the (dis)similarity between a sequence and its projection on sample *s*. The similarity function is formally defined as follows.

**Definition 17 .**

Given a gene sequence *o*_*i*_ and the gene sequence *S* corresponding to sample *s*, the* PD similarity* between *o*_*i*_ and *o*_*i*_∣*s*, denoted as Sim_PD_(*o*_*i*_, *o*_*i*_∣*s*), is defined as

(4) $$\begin{array}{c} \text{Si}{\text{m}}_{\text{PD}}\left(o_{i},o_{i}\;|\;s\right)=1-\frac{\text{PD}\left(o_{i},o_{i}\;|\;s\right)}{\sum_{j=1}^{o_{i}}\left(\left|o_{i}\right|+1-j\right)*\left(\left|o_{i}\right|-j\right)}, \end{array}$$

where |*o*_*i*_| is the length of gene sequence *o*_*i*_.

From (4), we can find that the smaller the projection distance between two sequences, the more the similarity of the sequences. If PD(*O*_1_, *O*_2_) = 0, Sim_PD_(*o*_1_, *o*_2_) = 1, which means the two sequences are totally the same. Next, we introduce the criteria of ranking rules with two cases.

**Definition 18 .**

*The priority within the same rule group*: given two rules *γ*_1_: *X*_1_ → *C*,  *γ*_2_: *X*_2_ → *C*, and *R*(*γ*_1_) = *R*(*γ*_2_), we say *γ*_1_ is prior to *γ*_2_ if

(5) ∑s′∈(S−R(γ1))SimPD(X1,X1 ∣ s′)S−Rγ1 <∑s′∈(S−R(γ2))SimPD(X2,X2 ∣ s′)S−Rγ2.

From (5), we can conclude that the more the antecedent of the rule is different from the gene sequence in the nonsupport set, the higher the priority the rule has.

**Example 19 .**

In Figure 2, the support sets of rules 〈*g*_3_ *g*_4_〉 → *C*_1_ and 〈*g*_7_ *g*_5_〉 → *C*_1_ are both {*s*_1_, *s*_2_, *s*_3_}, but based on (5), (0 + 0 + 0)<(0 + 0 + 1/3), so 〈*g*_3_ *g*_4_〉 → *C*_1_ is more prior than 〈*g*_7_ *g*_5_〉 → *C*_1_.

**Definition 20 .**

*The priority between rule groups*: given two rules *γ*_1_: *X*_1_ → *C*, *γ*_2_: *X*_2_ → *C*, and *R*(*γ*_1_) ≠ *R*(*γ*_2_), we say *γ*_1_ is prior to *γ*_2_, if and only if one of the following three conditions satisfied: (1)  conf(*γ*_1_) > conf(*γ*_2_); (2)  conf(*γ*_1_) = conf(*γ*_2_) and supp(*γ*_1_) > supp(*γ*_2_); (3)  conf(*γ*_1_) = conf(*γ*_2_), supp(*γ*_1_) = supp(*γ*_2_) and *γ*_1_ is discovered before *γ*_2_.

**Example 21 .**

In Figure 2, assume *γ*_1_: 〈*g*_3_ *g*_4_〉 → *C*_1_, *γ*_2_: 〈*g*_6_ *g*_5_ *g*_4_〉 → *C*_1_, and *γ*_3_: 〈*g*_7_ *g*_4_〉 → *C*_1_. Because conf(*γ*_1_) = conf(*γ*_2_) = 100% and supp(*γ*_1_) = 3, which is higher than that of supp(*γ*_2_) = 2, *γ*_1_ is more prior than *γ*_2_. Also, conf(*γ*_3_) = 75% and conf(*γ*_2_) > conf(*γ*_3_), so *γ*_2_ is more prior than *γ*_3_.

## 4. The MineTop*k*IRs Algorithm

In this section, we present our algorithm, called MineTop*k*IRs, to solve the problem given in Problem Statement. First, we give a naive method to construct classifier based on contrast sequence rules.

**Step 1 .**

Discover all the frequent sequence patterns with a low minimum support threshold.

**Step 2 .**

Combine each sequence pattern with a class label to generate a sequence rule. Then, pick out the contrast sequence rule with highest confidence for each sample in the dataset.

Obviously, this naive two-step mining method generates too many rules in Step 1, which takes too long time. Moreover, selecting only one rule for each sample is often not enough. Instead, our algorithm is one-pass process, which is much more efficient. Further, each sample is guaranteed to be covered by top-*k* irreducible contrast sequence rules. In what follows, we detail the proposed MineTop*k*IRs algorithm.

### 4.1. Head-Tail Matrix

The Head-Tail matrix *M* is a useful structure to accelerate the detection whether a sequence is a significant chain corresponding to some sample template sequence $_*i*_, which is a necessary condition of the antecedent of a contrast sequence rule.  Table 2 gives the Head-Tail matrix corresponding to the model shown in Figure 2, where each row represents a considered sample, and each column represents a remained gene. Every entry (*i*, *j*) in the matrix *M* records a two-dimensional vector (*x*, *y*), where *x* denotes the head position of the gene *g*_*j*_ in $_*i*_, that is, *H*_*i*_(*g*_*j*_), and *y* denotes the tail position of the gene *g*_*j*_ in $_*i*_, that is, *T*_*i*_(*g*_*j*_). For example, in Figure 2, *H*_3_(*g*_1_) = 2 and *T*_3_(*g*_1_) = 3, so the entry at row 3 and column 1 of Table 2 records (2,3).

An efficient way to decide whether a sequence *X* is a significant chain with respect to $_*i*_ is that we only consider any neighboring pair of genes such as *g*_*a*_ and *g*_*b*_; if *T*_*i*_(*g*_*a*_) < *H*_*i*_(*g*_*b*_) is always true, we say that *X* must be a significant chain for $_*i*_, which is the sequence of EDGs of sample *s*_*i*_.* Note*: While computing the support of a gene sequence, we use the Head-Tail matrix with *δ* > 0, which makes the order between genes in the sequence significant enough. However, when computing the projection distance of a gene sequence for some $_*i*_, we use the Head-Tail matrix with *δ* = 0, which makes the displacement of a reverse pair easily determined.

### 4.2. The Mining Algorithm

The search space of enumerating all gene sequences is prohitably large. Thus, a suitable traversal framework with some effective pruning strategies is necessary.

In this paper, we adopt a breadth-first traversal framework. As we know, most sequence pattern mining methods such as BIDE [9] and FEAT [10] adopt a depth-first traversal. The goodness is that exploiting the antimonotonicity of support, the depth-first traversal can directly prune searching space based on the current sequence without generating candidate set. However, depth-first traversal is not suitable to solve the problem raised in this paper. The reason is that (1) the confidence of irreducible contrast sequence rule is not antimonotonic, which requires us to detect whether all subrules of the current rule satisfy the conditions defined in Definition 12 that is the confidence of all subrules below *β*. For example, suppose the length of current sequence rule is *l*, we need to detect all the subrules, which shows the computation is very large. (2) Under the premise of not establishing access rules index, it is possible to repeatedly access many rules. The abovementioned two cases are very time-consuming. On the contrary, the breadth-first traversal can be a good solution to the problem mentioned above. We only need to detect whether all the (*l* − 1)-size subrules meet the conditions. Further these subrules can be obtained by directly accessing the current rule candidate set which is more efficient.

Formally, the algorithm is shown in Algorithm 1. There are four input parameters of the algorithm, the original dataset *D*, equivalent threshold *δ*, the minimum support *α*, and confidence threshold *β*. Because of solving the problem in gene sequence perspective, the algorithm will first transform *D* into the EWave model *D* and then construct the Head-Tail matrix which can accelerate the calculation of rule support. At the same time, the top-*k* covering irreducible contrast sequences rules for each sample *s*_*i*_ with consequent *C*, denoted as *ζ*_*s*_*i*__ = [*γ*_*s*_*i*_1_,…, *γ*_*s*_*i*_*k*_], will be initialized. Also, we put all the 1-size rules that consist of single gene into rule candidate set* Candi_R*. Then the function* breathfirst_search* is called to perform the breath-first traversal to find out the top-*k* rules for each sample.

The function* breathfirst_search* takes in four parameters: the rule candidate set* Candi_R*, minimum support *α*, confidence threshold *β*, and the size of rule *l*. When the algorithm executes to the *l* level, it generates all the (*l* + 1)-size rules based on the rules in* Candi_R* (line 2). For each (*l* + 1)-size rule, the algorithm is based on three pruning rules (lines 4, 6, and 11) to detect whether it will be put into* Candi_R* for further extension (line 8) or used to update the top-*k* covering rules for samples in its support set (line 13) or just be pruned. It is worth noting that the confidence of all the rules in* Candi_R* must be below *β* because once the confidence of a rule exceeds *β*, all the super rules of it cannot be irreducible contrast sequence rules. After the end of each loop, the algorithm deletes the whole* l*-size rules in* Candi_R* (line 17). The algorithm ends when* Candi_R* = *ϕ* (line 1).

#### 4.2.1. Pruning Strategies

We next illustrate the pruning techniques that are used in MineTop*k*IRs. With the help of these pruning rules, we can find out the top-*k* covering irreducible contrast sequence rules for each sample efficiently.

*Pruning Rule 1.* Let *γ*: *X* → *C* be the current considered sequence rule; if there exists a sequence rule *γ*′: *X*′ → *C*, *X*′⊑*X*, and conf(*γ*′) > *β*, the rule itself and all its super rules can be pruned.

**Proof**

Based on the definition of irreducible contrast sequence rule, if a sequence rule *γ*: *X* → *C* is irreducible contrast sequence rule, it requires that ∀*γ*′: *X*′ → *C*  (*X*′⊑*X*), conf(*γ*′) < *β*. Thus, if any of its subrules *γ*′: *X*′ → *C* do not satisfy this condition, the sequence rule *γ*: *X* → *C* cannot be an irreducible contrast sequence rule. Similarly, none of its super rules can be irreducible contrast sequence rules.

Specific to our algorithm, we store each rule whose confidence and all its subrules' confidence are below *β* in* Candi_R* for further extension. When deciding whether a newly generated *l*-size rule is to be pruned, we only need to test if all of its (*l* − 1)-size subrules are in* Candi_R*. If not, we can safely prune this sequence rule and all its super rules.

*Pruning Rule 2.* Let *γ*: *X* → *C* be the current considered sequence rule and *α* the minimum support threshold. If supp(*X* → *C*) < *α*, then the current rule *γ* and all its super rules are pruned.

**Proof**

It is immediately derived from the a priori property of sequence [11] and Definition 12.

In MineTop*k*IRs, we can use the constraint of top-*k* to prune rules. Combined with Definition 20, we compute minconf and sup, the critical point of Top*k*IRs thresholds for the samples in *R*(*γ*), where minconf is the minimum confidence value of the discovered Top*k*IRs of all the samples in *R*(*γ*) and sup is the corresponding support. Assume the top-*k* covering irreducible contrast sequence rules of each sample *s*_*i*_ are ranked according to the priority between rule groups such that *γ*_*s*_*i*_1_≺*γ*_*s*_*i*_2_≺⋯≺*γ*_*s*_*i*_*k*_:

(6) $$\begin{array}{c} \text{minconf}=\text{mi}{\text{n}}_{s_{i}\in R(\gamma )}\left\{\text{conf}\left(\gamma_{s_{i}k}\right)\right\},\\ \sup=\sup\left(\gamma_{s_{x}k}\right),\;\text{where}\;\;\text{conf}\left(\gamma_{s_{x}k}\right)=\text{minconf}. \end{array}$$

*Pruning Rule 3.* Given the current considered sequence rule *γ*: *X* → *C* and conf(*γ*) ≥ *β*, minconf and sup computed according to (6), if the rule is less prior based on the priority between rule groups (Definition 20) than *γ*_*s*_*x*_*k*_(conf(*γ*_*s*_*x*_*k*_) = minconf, sup⁡ = sup⁡(*γ*_*s*_*x*_*k*_)), then the rule *γ* and all its super rules cannot become a rule in the top-*k* covering irreducible contrast sequence rules list of any sample and can be safely pruned.

If the current sequence rule *γ*: *X* → *C* cannot be pruned by Pruning Rule 3, there are two situations. On one hand, ∀*s*_*i*_ ∈ *R*(*γ*) when there are no rules in {*γ*_*s*_*i*_1_,…, *γ*_*s*_*i*_*k*_} that have the same sample support set as that of *γ*, we only need to detect if *γ* is more prior than *γ*_*s*_*i*_*k*_, if so, we substitute *γ*_*s*_*i*_*k*_ for *γ*. On the other hand, because in this paper we want to find out top-*k* rules for each sample with different sample support sets, ∀*s*_*i*_ ∈ *R*(*γ*) when there is some rule in {*γ*_*s*_*i*_1_,…, *γ*_*s*_*i*_*k*_} that has the same sample support set as that of *γ*, we need to find out that if *γ* is more prior than the rule has the sample support set with *γ* based on the priority within the same rule group (Definition 18), if so, we replace this rule with *γ* which can guarantee the current rules in {*γ*_*s*_*i*_1_,…, *γ*_*s*_*i*_*k*_} have the highest priority.

In addition, another optimization method is utilized in Pruning rule 3. If we find all Top*k*IRs have 100% confidence and the lowest support value of *k* rules is larger than *α*, we dynamically increase the user-specified support threshold.

## 5. Performance Studies

In this section, we will look at both the efficiency of our algorithm in discovering Top*k*IRS and the usefulness of the discovered rules. All our experiments were performed on a HP PC with 2.33 GHz Intel Core 2 CPU, 2 GB RAM, and a 160 GB hard disk running Windows XP. Algorithms were coded in Standard C.

*Datasets.* Four real gene expression datasets for experimental studies: Leukemia [1], DLBCL Tumor [2], Hereditary Breast Cancer (HBC) [3], and Prostate Cancer (PC) [12].  Table 3 shows the characteristics of the four datasets: the number of samples (#sample), the number of genes (#gene), and the label of class *i*  (*C*_*i*_). The number of samples in every class is shown in the last column. Moreover, we generate the synthetic datasets by using a specialized dataset generator [8].

### 5.1. Efficiency of MineTop*k*IRs

In term of efficiency, we compare MineTop*k*IRs with R-FEAT and NRMINER [8]. On one hand, R-FEAT is changed from the sequence generator mining algorithms FEAT [10]. Briefly, we apply FEAT on a given dataset, when a generator *X* is found, we decide whether *X* → *C* could be a result by checking all rules *X*′ → *C*, where *X*′⊑*X*, satisfying the conditions based on Definition 14. On the other hand, the NRMINER algorithm adopts the template driven method to find out all the interesting nonredundant contrast sequence rules, which are necessary for checking whether the conditions in Definition 14 are satisfied. We should point out that the rules discovered by MineTop*k*IRs are a subset of the above two existing methods.

**Figure 3:**
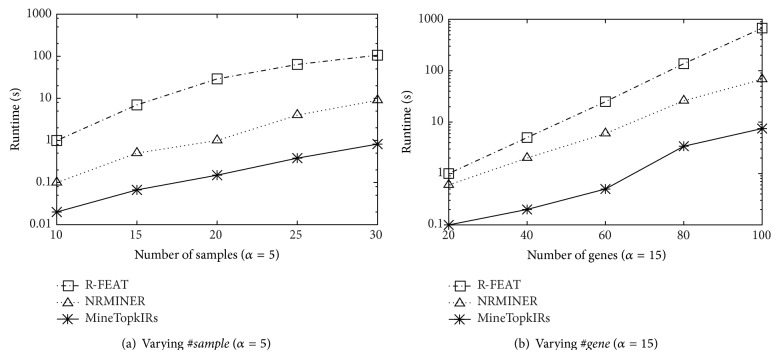
Scalability.

In Figure 3, we study how the running time varies with #*sample* and #*gene* by increasing #*sample* from 10 to 30 while fixing #*gene* to 100 and then increasing #*gene* from 20 to 100 while fixing #*sample* to 30, where the synthetic datasets are utilized. Figures 3(a) and 3(b) show that the running time becomes longer with #*sample* and #*gene* increasing. This is because the searching space also becomes larger. However, the MineTop*k*IRs is always much faster than the other two methods; the reason is that our algorithm can directly discover the results in one step. However, the other two are two-step mining methods, which need to first discover a bigger result set and then conduct the postprocessing. Further, with the searching space increasing, the number of rules after first step mining grows exponentially. Thus, it is very time-consuming.

**Figure 4:**
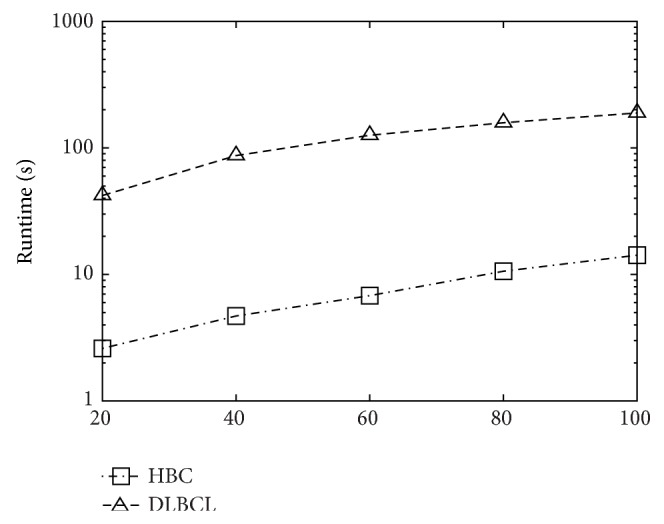
Varying *k*.

**Figure 5:**
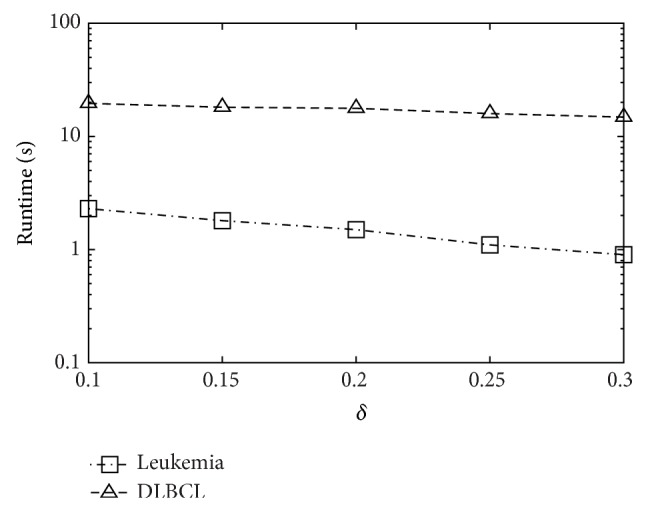
Varying *δ*.

**Figure 6:**
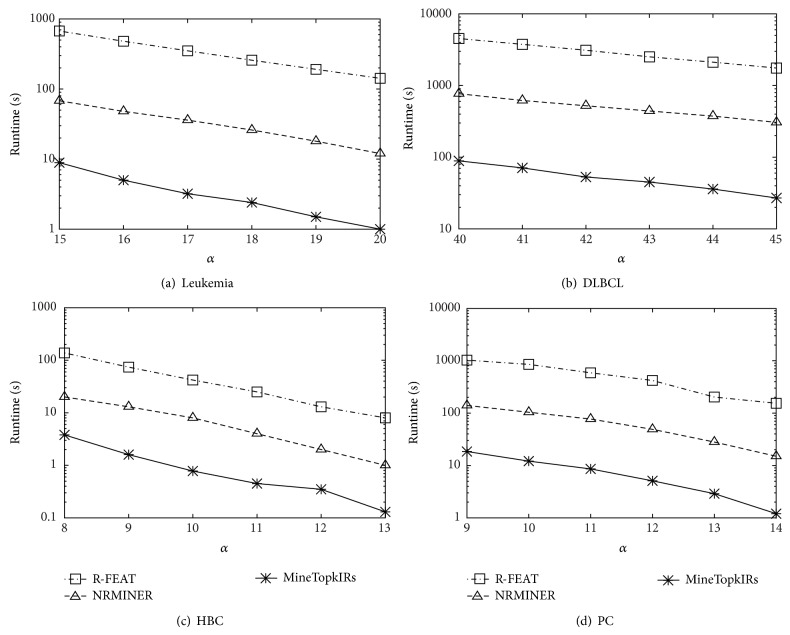
Varying *α*.

**Figure 7:**
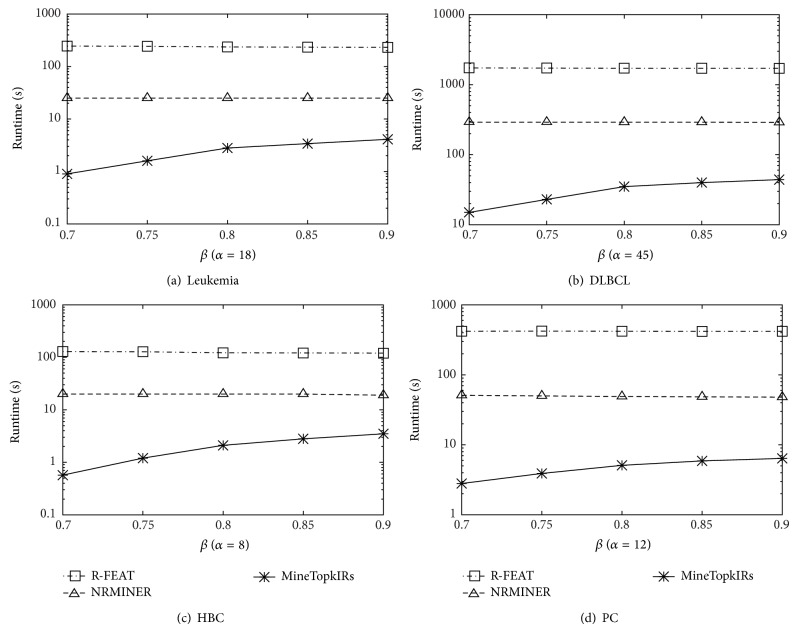
Varying *β*.

Figure 4 shows the effect of varying *k* towards runtime. We observe similar tendencies on all datasets. It is quite reasonable that MineTop*k*IRs is monotonously increasing with *k*. Also, as shown in Figure 5, MineTop*k*IRs is monotonously decreasing with *δ*. Figures 6 and 7 show the effect of varying minimum support threshold *α* and the minimum confidence threshold *β* on four real gene expression datasets. Figures 6(a)–6(d) show the running time varying with the minimum support threshold *α*, where the other two parameters *β* and *δ* are set to 0.8 and 0. Note that the *y*-axes in Figures 6 and 7 are in logarithmic scale. We run MineTop*k*IRs by setting *k* = 10. In Figure 7, *β* changes from 70% to 90% while *δ* = 0 and *α* is fixed in every dataset. As seen from Figure 6, running time decreases with the increasing of *α*. This is because the increasing of *α* prunes more useless rules. We also find out that MineTop*k*IRs is usually one order of magnitude faster than the other two algorithms, especially at low minimum support. The reason MineTop*k*IRs outperforms the other two algorithms is that R-FEAT and NRMINER discover a large number of rules at lower minimum support while the number of rules discovered by MineTop*k*IRs is bounded. Besides, MineTop*k*IRs can use Pruning strategy 1 to prune the search space; however, R-FEAT and NRMINER do not meet this property. Figure 7 shows that the running time of both NRMINER and R-FEAT does not change significantly as *β* is increasing, which is because the pruning strategies of these methods are mainly based on support threshold *α*. However, the running time a little increases with the increasing *β*. This is because with the increasing of *β*, the rules whose confidence below *β* will also increase; thus the pruning ability decreases a little. Despite so, the MineTop*k*IRs is still faster than the other two algorithms based on the above reasons in Figure 6.

### 5.2. Effectiveness of MineTop*k*IRs

In terms of the effectiveness of MineTop*k*IRs, the classification accuracy and the complexity are used as the performance standard for evaluation. Moreover, the biological significance of the discovered genes is also discussed.

#### 5.2.1. Accuracy and Complexity

We build a classifier called Top*k*IR classifier based on the rules that MineTop*k*IRs discovered. The Top*k*IR classifier is composed of *k* subclassifiers, denoted as IR_1_,…, IR_*k*_. Each IR_1_ classifier is built based on all the top-*j* rules for each sample in the dataset. We call IR_1_ the main classifier and IR_2_,…, IR_*k*_ are backup classifiers. We use every subclassifier in order until the test sample is successfully classified. Besides both main and backup classifiers we set a default class which is set as the majority class of the training data. If a test sample cannot be classified by the *k* classifiers, we put it into the default class.

When building each subclassifier, the score function in (7) [13] is adopted, where *r* ∈ *ℛ*(*C*, *s*) represents the rules matching the test sample *s* in class *C*, and *r* ∈ *ℛ*(*C*) represents all the rules in class *C*. To which class a test sample should be assigned is decided by a matched rule of the highest score:

(7) $$\begin{array}{c} \text{Score}\left(s\in C\right)=\frac{\sum_{r\in R(C,s)}\mathrm{supp}\left(r\right)\text{conf}\left(r\right)}{\sum_{r\in R(C)}\mathrm{supp}\left(r\right)\text{conf}\left(r\right)}. \end{array}$$

In the experiments, we adopt 10-fold cross validation to test the average classification accuracy of Top*k*IR classifier and compare it with NR [8] and CBA and IRG [14] classifiers. The results in Table 4 show that Top*k*IR classifier performs much better than CBA and IRG classifiers. Compared with CBA which is built with the Top-1 covering irreducible contrast sequence rules, Top*k*IR classifies much fewer test data using default class. IRG classifier is built based on the association rules, which illustrates that sequence rules can reflect data characteristics better. Top*k*IR classifier is more accurate than NR classifier on most dataset; however, it uses much fewer rules (*m*∗*k*) to build classifier than NR. In our experiment, *k* = 10 and the rules used in NR classifier are usually more than ten thousand [8]. Furthermore, we discover that the average length (AL for short) of sequence rules used in Top*k*IR classifier is shorter than that of IRG. This result verifies that the MineTop*k*IRs could provide as high as diagnostic accuracy using as fewer as possible genes, which is very valuable for biologists to further follow up biological or clinical validation of selected genes [15].

#### 5.2.2. Biological Significance

Different from the traditional methods, MineTop*k*IRs characterizes the pathogenesis of a disease from a sequence-like point of view, which incorporates the orders among genes and can be seen as the pathway of disease causing. In this part, by showing some interesting results from Leukemia dataset [1], we emphasize the fact that not only can MineTop*k*IRs find the genes revealed by the traditional methods, but also it can find some genes ignored by the traditional methods.

Table 5 lists the top-10 genes most frequently occurring in the discovered Top*k*IRs for the diagnosis of “AML” samples and “ALL” samples, where the genes with “∗” mean they are also included in the benchmark, that results from eight statistics based gene ranking methods [16]. The two most frequent genes appear in Table 5, which also appear in the benchmark. Gene TIMP2 is a member of the TIMP gene family, the proteins encoded by which are natural inhibitors of the matrix metalloproteinases. Reference [17] reveals that the transcription of TIMP2 in SHI-1 cells of AML is higher than other leukemic cells. Gene ZFP36 expression is upregulated in human T-lymphotropic virus 1- (HTLV-1-) infected cells. HTLV-1 is associated with adult T-cell leukemia/lymphoma [18].

In addition, for the genes without “∗”, though they are not in the benchmark, we still cannot ignore these genes. For example, the gene sequence 〈*RBL*2  *DHPS* *CCT*5〉 including frequent gene CCT5 in Table 5 appears in most “ALL” sample but fewer occurs in “AML” samples. But, any of its subsequence does not have the ability of distinguishing samples which indicates that any gene in 〈*RBL*2  *DHPS* *CCT*5〉 is irreducible and well reflects the synergy between the genes. Thus, these genes also have very important potential values for biologists to further explain.

## 6. Conclusion

In this paper, we study an important problem in bioinformatics, that is, discovering diagnostic gene patterns from gene expression data. Unlike any previous work on this topic, we tackle the problem by exploiting the ordered expression trend of genes, which can better reflect the gene regulation pathway. In order to capture the more accurate diagnosis by using as few as possible rules, we propose the concept of top-*k* covering irreducible contrast sequence rules for each sample of gene expression data. Further, an efficient method called MineTop*k*IRs is developed to find all Top*k*IRs. Considering the real noisy scenario in gene expression data, we first use an EWave model, which, essentially different from the current models, characterizes gene expression data from a sequence-like perspective. Then, we can use MineTop*k*IRs to discover the bounded number of Top*k*IRs in one mining process, which can directly be used to build classifier. Extensive experiments conducted on both synthetic and real datasets show that MineTop*k*IRs is both effective and efficiency. It may offer a new point of view from diagnostic gene discovery to the biologists.

## Figures and Tables

**Algorithm 1:**
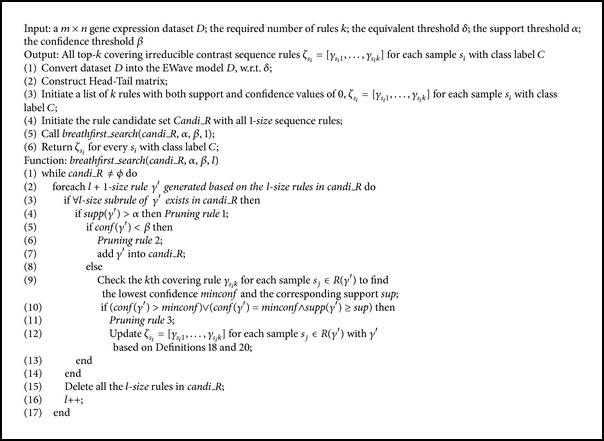
The MineTop*k*IRs Algorithm.

**Table 1:** An illustrative expression matrix with sample labels.

Sample	g_1_	g_2_	g_3_	g_4_	g_5_	g_6_	g_7_	tag
*s*_1_	2.2	1.3	0.8	2.38	1.44	0.3	0.48	*C*_1_
*s*_2_	3.3	1.25	2.54	6.3	2.3	0.62	1.4	*C*_1_
*s*_3_	1.26	6.6	3.1	5.4	5.62	0.94	1.72	*C*_1_
*s*_4_	4.3	0.34	7.2	7.1	1.9	2.1	2.66	*C*_2_
*s*_5_	2.78	0.62	5.1	1.86	1.74	1.34	2.92	*C*_2_
*s*_6_	1.1	2.85	2.1	0.48	2.4	0.52	2.33	*C*_2_

**Table 2:** The Head-Tail matrix of gene expression data.

Sample	g_1_	g_2_	g_3_	g_4_	g_5_	g_6_	g_7_	tag
*s*_1_	5.5	4.4	3.3	5.5	4.4	1.1	2.2	*C*_1_
*s*_2_	3.3	2.2	3.3	4.4	3.3	1.1	2.2	*C*_1_
*s*_3_	2.3	1.1	4.4	5.5	5.5	2.2	3.3	*C*_1_
*s*_4_	3.3	1.1	4.4	4.4	2.2	2.2	2.2	*C*_2_
*s*_5_	3.3	1.1	4.4	4.4	2.2	2.2	2.2	*C*_2_
*s*_6_	2.2	3.3	3.3	1.1	3.3	1.1	3.3	*C*_2_

**Table 3:** The information of gene expression data.

Dataset	# sample	# gene	*C*_1_	*C*_2_	*C*_1_ : *C*_2_
Leukemia	38	5000	ALL	AML	27 : 11
DLBCL	77	7129	DLBCL	FL	58 : 19
HBC	22	3326	BRCA	Sporadic	15 : 7
PC	25	6500	Cancer	BPH	16 : 9

**Table 4:** The accuracy and complexity of classifiers.

Dataset	Acc. of Top*k*IR	Acc. of NR	Acc. of CBA	Acc. of IRG	AL. of Top*k*IR	AL. of Top*k*IR
Leukemia	96.84%	95.76%	91.18%	64.71%	3.42	4.80
DLBCL	94.97%	92.13%	82.16%	84.42%	3.16	4.95
HBC	92.68%	93.31%	85.61%	83.28%	2.45	4.25
PC	96.06%	91.28%	84.65%	88.24%	3.85	5.25

**Table 5:** Common frequent genes appear in Top*k*IRs.

Frequent gene in Top*k*IRs	Frequency (%)	Frequent gene in Top*k*IRs	Frequency (%)
TIMP2^*^	19.2	PTPRCAP^*^	7.9
ZFP36^*^	12.5	CCT5	5.6
MGST1^*^	12.4	CMYB	5.2
MYCL1	11	PSMA6^*^	4.9
LYZ1^*^	10.6	GIRX^*^	4.6
